# Periostin down-regulation attenuates the pro-fibrogenic response of hepatic stellate cells induced by TGF-β1

**DOI:** 10.1111/jcmm.12636

**Published:** 2015-08-07

**Authors:** Li Hong, Dai Shejiao, Chen Fenrong, Zhao Gang, Dong Lei

**Affiliations:** a Department of Gastroenterology, The Second Affiliated Hospital of Xi’an Jiaotong UniversityXi’an, Shaanxi, China

**Keywords:** periostin, hepatic stellate cells, fibrosis, TGF-β1

## Abstract

Liver fibrosis is characterized by an exacerbated accumulation of deposition of the extracellular matrix (ECM), and the activation of hepatic stellate cells (HSC) plays a pivotal role in the development of liver fibrosis. Periostin has been shown to regulate cell adhesion, proliferation, migration and apoptosis; however, the involvement of periostin and its role in transforming growth factor (TGF)-β1-induced HSC activation remains unclear. We used RT-PCR and Western blot to evaluate the expression level of periostin in hepatic fibrosis tissues and HSCs, respectively. Cell proliferation was determined using the Cell Proliferation ELISA BrdU kit, cell cycle was analysed by flow cytometry. The expression of α-smooth muscle actin (α-SMA), collagen I, TGF-β1, p-Smad2 and p-Smad3 were determined by western blot. Our study found that periostin was up-regulated in liver fibrotic tissues and activated HSCs. In addition, siRNA-periostin suppressed TGF-β1-induced HSC proliferation. The HSC transfected with siRNA-periostin significantly inhibited TGF-β1-induced expression levels of α-SMA and collagen I. Furthermore, TGF-β1 stimulated the expression of periostin, and siRNA-periostin attenuated TGF-β1-induced Smad2/3 activation in HSCs. These results suggest that periostin may function as a novel regulator to modulate HSC activation, potentially by promoting the TGF-β1/Smad signalling pathway, and propose a strategy to target periostin for the treatment of liver fibrosis.

## Introduction

Hepatic fibrosis represents a frequent event following chronic insult to trigger wound healing responses in the liver. Hepatic fibrosis, the excessive deposition of the extracellular matrix (ECM), is the main complication of chronic liver disease, such as viral infection, autoimmune hepatitis, alcohol consumption, biliary obstruction, non-alcoholic fatty liver disease and loss of parenchymal tissue, resulting in pathophysiological damage to the organ 1. Despite an increase in knowledge of the pro-fibrotic pathways leading to pathological fibrosis, the appropriate strategies to treat hepatic fibrosis have not been well-established.

It is widely recognized that hepatic stellate cells (HSC) are the most relevant cell type for the development of liver fibrosis, and their activation plays a pivotal role in the process of liver fibrosis. In the healthy liver, HSC have a quiescent phenotype and are mainly responsible for the uptake, storage and delivery of retinoids. After liver injury, these quiescent HSC are exposed to pro-fibrogenic factors or reactive oxygen species, and undergo a process of activation to a myofibroblastic phenotype, finally resulting in the excess production and deposition of ECM components, which are responsible for scarring and are involved in a series of inflammatory and fibrotic processes 2. As a variety of factors regulate collagen synthesis and turnover by a number of cells, developing strategies to interfere with fibrosis has been a significant challenge 3.

Periostin, also known as osteoblast-specific factor 2, is a recently characterized matricellualr protein that binds to the components of the ECM, including type I collagen and fibronectin, and has been involved in collagen fibrillogenesis 4. Also, this protein transmits signals from the ECM to the cell by binding to cellular receptors to affect cell functions, such as cell adhesion, proliferation, migration and tissue angiogenesis 5. Its expression in healthy adult tissues is very low but can be up-regulated by mechanical stress 6,7. Periostin has been found to be involved in the healing process after myocardial infarction by inducing the proliferation of cardiomyocytes, or after wound development by activating keratinocytes and fibrosis 8,9. It has been reported that periostin promotes cancer cell invasion and metastasis *via* the integrin/phosphatidylinositol 3-kinase/Akt pathway, leading to the development of various tumours 10. However, little is known about the role of periostin in hepatic fibrosis. In this study, the role of periostin in liver fibrosis was investigated. We found that siRNA-periostin attenuated liver fibrosis by inhibiting the transforming growth factor (TGF)-β1/Smad signalling pathway.

## Material and methods

### Specimen collection

Liver biopsies were collected by transparietal puncture from 8 healthy individuals and 10 patients with liver fibrosis, diagnosed on clinical, biological and histological grounds. Plasma was collected by centrifuging the blood samples at room temperature and thereafter stored at −80°C. All participants provided written informed consent, and the specimen collection procedure was approved by the Medical Ethics Committee of the Second Affiliated Hospital of Xi’an Jiaotong University.

### Cell culture

HSC-T6 cells, which were purchased from the American Type Culture Collection (Manassas, VA, USA), were grown in DMEM supplemented with 10% heat-inactivated foetal bovine serum at 37°C in a humidified 5% CO_2_ atmosphere. The cultures were passaged by trypsinization every 3 days.

### siRNA transfection

siRNA-periostin and siRNA-scramble were purchased from Cell Signaling (Beverly, MA, USA). For transfection, 5 × 10^4^ cells were seeded in each cell of a 24-well micro-plate, grown for 24 hrs to reach 30–50% confluence, and then incubated with a mixture of siRNA and Lipofectamine 2000 reagent (Invitrogen, Carlsbad, CA, USA) in 100 μl serum-free DMEM, according to the manufacturer’s instructions. The transfection efficiency was examined by Real-Time PCR and Western blotting.

### Real-time PCR

Total RNA was extracted using Trizol Reagent according to the manufacturer’s instructions (Invitrogen). Then, 2 μg of total RNA was transcribed to first-strand cDNA using TaqMan reverse transcription reagents (Applied Biosystems, Foster City, CA, USA). The following primers were used: periostin, 5′-GGGGTTGTCACTGTGAACTG-3′ (sense), 5′-CGGCTGCTCTAAATGATGAA-3′ (antisense) periostin (Human) 5′-GAACCAAAAATTAAAGTGATTGAAGG-3′ (sense), 5′-TGACTTTTGTTAGTGTGGGTCCT-3′ (antisense); α-smooth muscle actin (SMA), 5′-TGGCCACTGCTGCTTCCTCTTCTT-3′ (sense), 5′-GGGGCCAGCTTCGTCATACTCCT-3′ (antisense); collagen I, 5′-TGACTGGAAGAGCGGAGAGTACT-3′ (sense), 5′-GCTGTGGGCATATTGCACAA-3′ (antisense); collagen I (Human) 5′-TCTGGAGAGGCTGGTACTGC-3′ (sense), 5′-GAGCACCAAGAAGACCCTGA-3′ (antisense) and β-actin 5′-CCGTGAAAAGATGACCCAGATC-3′ (sense), 5′-CACAGCCTGGATGGCTACGT-3′ (antisense). Reactions were carried out using the Step One Plus real-time PCR machine (Applied Biosystems, Carlsbad, CA, USA).

### Western blot

Total protein was extracted from liver tissue or HSCs, then washed with ice-cold PBS and lysed with RIPA Cell Lysis Buffer (Cell Signaling) containing a phosphatase inhibitor and the protease inhibitor cocktail (Sigma-Aldrich, St. Louis, MO, USA), by incubating on ice for 30 min. Lysates were collected by centrifugation and protein concentrations were determined by the Bicinchoninic Acid (BCA) method. The samples (30 μg protein/lane) were separated on 10% SDS-PAGE and transferred onto polyvinylidene fluoride membranes. After blocking in Tris Buffered Saline (TBS) buffer (50 mmol/l NaCl, 10 mmol/l Tris, pH 7.4) containing 5% non-fat milk, the blots were incubated with primary antibodies (anti Periostin, anticollagen, anti-α-SMA, anti-p-Smad2, anti-p-Smad3, Smad2/3 or β-actin; Invitrogen) at 4°C overnight. Membranes were then washed and incubated with horseradish peroxidase-conjugated secondary antibodies. The blots were visualized by super ECL Western Blotting Substrate Kit and quantified by the Quantity ONE (Bio-Rad, Hercules, CA, USA) software. β-actin was used as the internal control.

### Cell proliferation assay

Cell proliferation was confirmed using the Cell Proliferation ELISA BrdU kit (Takara, Dalian, China). Briefly, the cells were plated at a density of 1.0 × 10^4^/well in a 24-well tissue culture plate. After incubation in serum-free media for 24 hrs, cells were treated with siRNA-periostin and stimulated with 10 ng/ml TGF-β1 simultaneously. After 48 hrs, the cells were incubated in the presence of BrdU at 37°C in a humidified incubator containing 5% CO_2_ for 24 hrs. Experiments were performed in triplicate in three independent experiments.

### Analysis of cell cycle

Cells were treated with siRNA-periostin before harvesting and fixing in ice-cold 70% ethanol for 1 hr. Cells were then washed with PBS and incubated in propidium iodide staining buffer (50 μg/ml propidium iodide, 0.1 mg/ml DNase-free RNase A and 0.5% Triton X-100) for 30 min. at 37°C in the dark. The DNA content was analysed by flow cytometry.

### Statistical analysis

Results were presented as mean ± SD. Statistical analysis was performed with one-way anova analysis. Statistical significance was determined at the level of *P* < 0.05.

## Results

### Periostin expression in hepatic fibrosis tissues and HSCs

**Figure 1:**
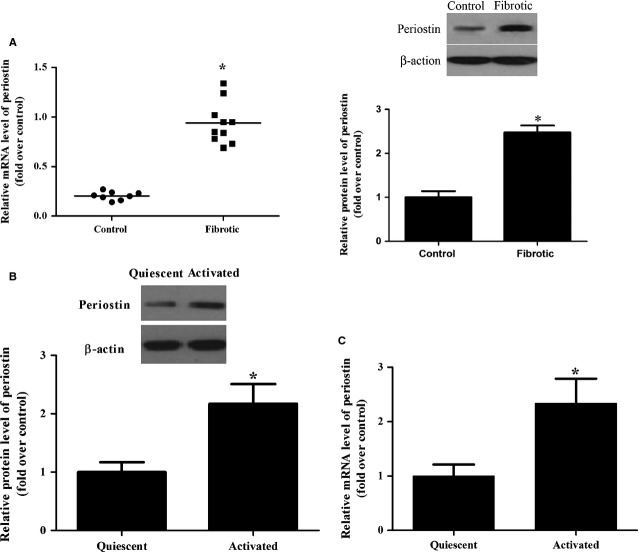
Periostin is up-regulated in hepatic fibrosis tissues and HSCs. (A) RNA was harvested from healthy (*n* = 8) and fibrotic (*n* = 10) human liver tissue, and the mRNA and protein expression of periostin were assessed by qRT-PCR and western blots, *P < 0.05 *versus* control; (B) represent western blots of periostin in quiescent and activated HSCs; (C) representative images of relative mRNA level of periostin in quiescent and activated HSCs. The expression levels of proteins were normalized based on the β-actin levels. *P < 0.05 *versus* quiescent.

To verify the expression of periostin in hepatic fibrosis, we determined the protein and mRNA levels of periostin in hepatic fibrosis tissues and HSCs. As shown in Figure 1A, the periostin mRNA and protein expression in hepatic fibrosis tissues was increased significantly compared to that of controls (*P* < 0.05). Similarly, we found that the protein and mRNA expressions of periostin were higher in HSCs than in the quiescent cells (Fig. 1B and C), indicating that aberrant periostin expression is a general feature of hepatic fibrosis.

### Periostin efficient down-regulation in HSCs by specific siRNA constructs

**Figure 2:**
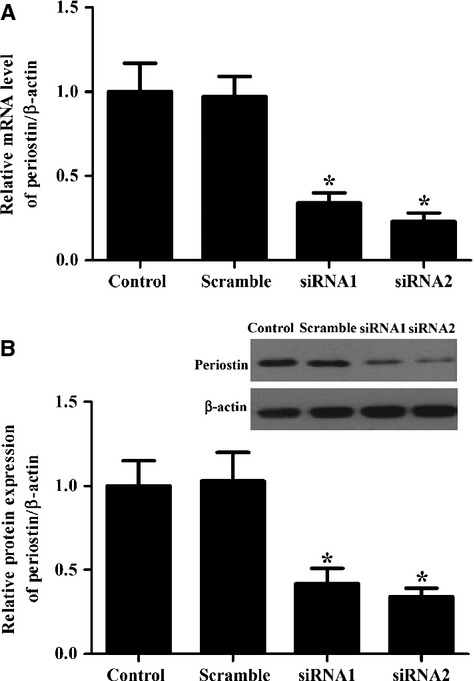
SiRNAs effectively inhibit periostin expression in activated HSCs. (A) Periostin mRNA expression was detected by qRT-PCR 48 hrs after transfection with siRNAs-periostin; (B) Western blot analysis of periostin expression 48 hrs after transfection with siRNAs-periostin. β-actin was used as a loading control. All experiments were repeated at least three times and all data are reported as means ± SD (*n* = 3; *P < 0.05 *versus* control).

To investigate the mechanisms involved in the anti-fibrotic effects of periostin, specific siRNA constructs were designed to silence periostin in HSCs. The efficiency of siRNA transfection was measured by RT-PCR and Western blot. As shown in Figure 2, siRNAs significantly decreased the expression levels of periostin. siRNA2-periostin was chosen for its high efficiency of transfection.

### Effect of periostin on proliferation and the cell cycle in activated HSC

**Figure 3:**
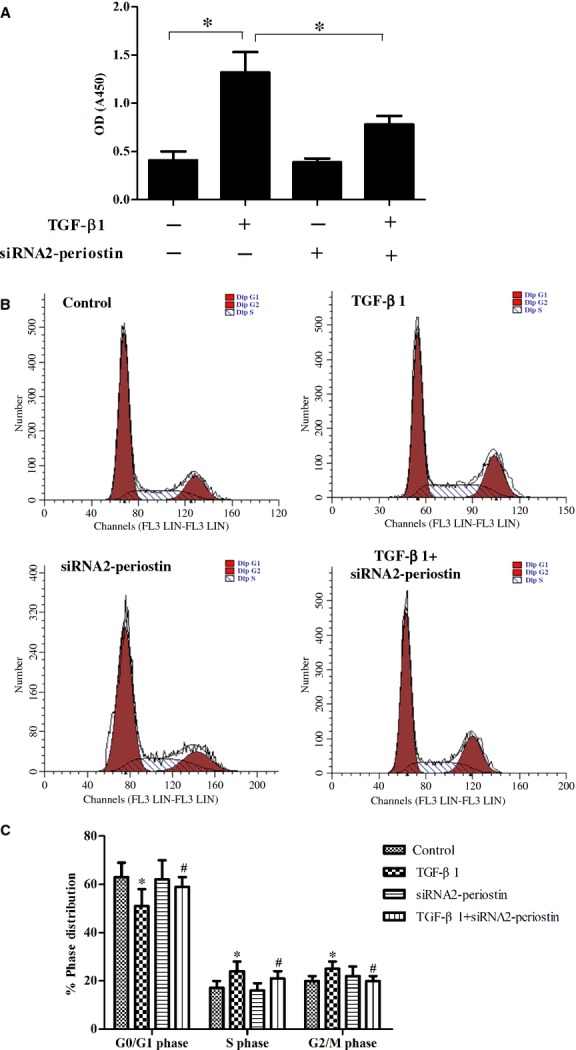
Effect of periostin on proliferation and the cell cycle in activated HSC. (A) siRNA-periostin reversed TGF-β1-increased BrdU incorporation; (B) cell cycle progression was evaluated by flow cytometry; (C) percentages of cell cycle distribution among different groups are presented as a histogram graph. The experiments were repeated three times and the data are shown as means ± SD. *P < 0.05 *versus* control; ^#^*P* < 0.05 *versus* TGF-β1.

Proliferation is one of the key behavioural changes of HSCs in the sustained activation state; therefore, we performed a BrdU-incorporation assay to investigate the effect of periostin on HSC proliferation. As shown in Figure 3A, compared with the control, the BrdU incorporation was obviously increased by TGF-β1, while siRNA-periostin reversed this effect (*P < 0.05).

To explore the possible mechanism underlying the anti-proliferation activity of siRNA-periostin, the cell cycle was determined by flow cytometry. As shown in Figure 3B and C, siRNA-periostin treatment promoted a decrease in the percent of cells in S phase and a corresponding increase in the percent of cells in G1 phase in TGF-β1-treated HSCs compared with the TGF-β1 group.

### Periostin down-regulation reduces the expression levels of α-SMA and collagen I in HSCs

**Figure 4:**
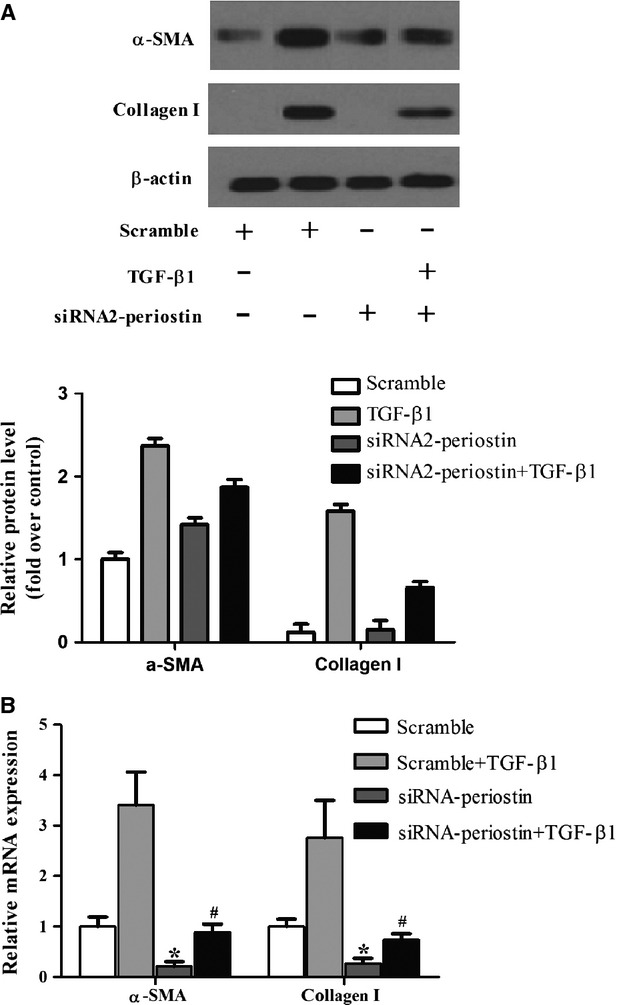
Periostin down-regulation reduces the expression levels of α-SMA and collagen I in HSCs. (A and B) The protein and mRNA levels of α-SMA and collagen I in different treatment groups. All experiments were repeated at least three times. *P < 0.05 compared with scramble; ^#^*P* < 0.05 compared with scramble+TGF-β1.

Periostin directly interacts with components of the ECM, including collagen and fibronectin, and promotes collagen cross-linking and fibrogenesis 4,11. Previous studies have shown that periostin can regulate collagen expression in osteoblasts and in fibrosis from various tissues 12,13. Therefore, we examined the effect of siRNA-periostin on α-SMA and collagen I expression levels in HSCs. As shown in Figure 4, siRNA-periostin dramatically decreased α-SMA and collagen I expression.

### Periostin is involved in the regulation of TGF-β1-mediated signalling pathways in HSCs

**Figure 5:**
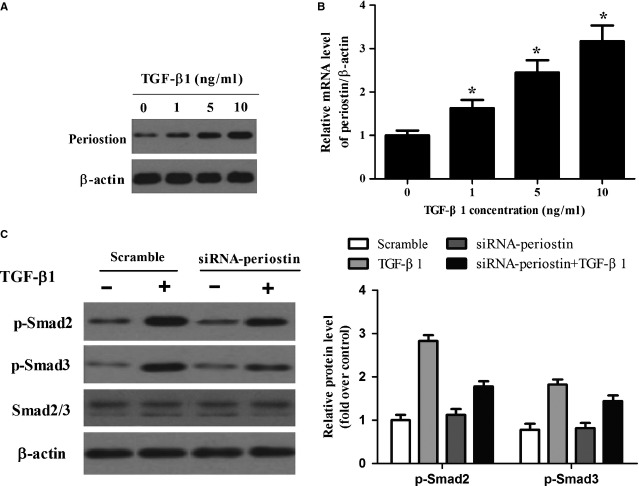
Periostin is involved in the regulation of TGF-β1-mediated signalling pathways in HSCs. (A and B) Concentration-dependent induction of periostin protein and mRNA by TGF-β1; (C) HSC-T6 cells were incubated with siRNA-periostin or non-target control for 96 hrs. Then cells were treated with TGF-β1 (10 ng/ml). The expression of p-Smad2, p-Smad3 and Smad2/3 were detected by Western blot analysis. Results are means ± SD from three independent experiments performed in duplicate. Relative expression is expressed in arbitrary units. *P < 0.05 compared with control.

Transforming growth factor-β1 is considered an important mediator of hepatic fibrosis. Periostin was also involved in HSC activation. Therefore, we examined the effect of periostin on TGF-β1-induced pro-fibrogenic signalling in HSCs. As shown in Figure 5, exposure of HSCs to TGF-β1 resulted in a dose-dependent increase in periostin expression. Transforming growth factor-β1 (1 ng/ml) slightly increased the protein levels of periostin, which was markedly increased at concentrations of 5.0 and 10.0 ng/ml periostin (Fig. 5A). Moreover, consistent with the protein level, the level of periostin mRNA was also increased by TGF-β1 (Fig. 5B). Furthermore, inhibition of periostin expression attenuated TGF-β1-induced Smad2/3 activation (Fig. 5C).

## Discussion

Liver fibrosis and its end-stage manifestation of cirrhosis represent clinical challenges worldwide. Hepatic stellate cells activation is the main feature that results in liver fibrosis; these activated HSCs increase proliferation and migration, and acquire contractility and pro-inflammatory properties. Therefore, anti-fibrotic therapeutic strategies include the inhibition of HSC proliferation, the down-regulation of collagen production and the promotion of collagen degradation 14. In this study, our results showed the anti-fibrogenic effects of siRNA-periostin on hepatic fibrosis.

Periostin plays an important role in cardiac development and remodelling and its distribution and expression are consistent with the extent of myocardial fibrosis 15. Another study reported that periostin^−/−^ mice on a C57Bl/6 background were protected from bleomycin-induced fibrosis compared with littermate controls 16. Recently, one report showed that the median serum periostin was significantly increased in hepatocellular carcinoma patients compared to healthy controls 17. In this study, the obvious up-regulation of periostin was observed in cirrhotic liver tissues and activated HSCs, which suggested that periostin may serve as a potential biomarker for hepatic fibrosis.

The induction of proliferation is an early step after HSC activation and is stimulated by a variety of cytokines 18. Transforming growth factor-β1 is the most potent stimulus for HSC-mediated fibrogenesis 19. PDGF induces the activation of several signalling pathways, such as Mitogen-activated protein kinases (MAPKs) and Akt, which have been reported to be involved in the modulation of HSC proliferation and migration during the progression of liver fibrosis 20. In this study, we found that siRNA-periostin suppressed TGF-β1-induced HSC proliferation.

Activated HSCs are the principal cell type that promotes the deposition of ECM proteins. Therefore, to investigate siRNA-periostin on the expression of α-SMA and collagen I, we transfected siRNA-periostin into HSCs followed by exposure to 10 ng/ml TGF-β1. Our findings demonstrated that siRNA-periostin reversed the up-regulation of α-SMA and collagen I under TGF-β1 stimulation. It has been reported that periostin enhances fibrosis by binding to other ECM proteins, and by inducing collagen fibrillogenesis through activating lysyl oxidase in the intramolecular cross-linking of collagen. This notion is supported by our results.

Activation of the TGF-β pathway is considered a key event in the development of liver fibrosis. Disruption of TGF-β signalling impedes HSC activation and fibrosis, as demonstrated by the altered expression of transdifferentiation markers 21. It has been reported that mice lacking the periostin gene expression showed inhibited TGF-β-pSmad3 signalling and are protected against inflammation and fibrosis. In addition, in previous studies, results have shown that TGF-β is a major stimulator of periostin secretion in a variety of tissues or cells, whereas the inhibition of TGF-β is accompanied by decreased periostin expression 22,23. In this study, we found that TGF-β1 increased the level of periostin, and inhibition of periostin expression attenuated TGF-β1-induced Smad2/3 activation in HSCs. These results suggested that siRNA-periostin attenuated liver fibrosis by inhibiting the TGF-β1/Smad signalling pathway.

In conclusion, these results suggest that periostin may function as a novel regulator to modulate HSC activation, potentially by promoting the TGF-β1/Smad signalling pathway, and propose a strategy to target periostin for the treatment of liver fibrosis.
